# Editorial Note: Access to and challenges in water, sanitation, and hygiene in healthcare facilities during the early phase of the COVID-19 pandemic in Ethiopia: A mixed-methods evaluation

**DOI:** 10.1371/journal.pone.0327891

**Published:** 2025-07-10

**Authors:** The PLOS One Editors

This article [1] was identified as one of a series of articles for which the *PLOS One* Editors have concerns about authorship and adherence to the journal’s first and second publication criteria. We regret that the issues were not addressed prior to the article’s publication.

## References

[1] Berihun G, Adane M, Walle Z, Abebe M, Alemnew Y, Natnael T, et al. Access to and challenges in water, sanitation, and hygiene in healthcare facilities during the early phase of the COVID-19 pandemic in Ethiopia: A mixed-methods evaluation. PLoS One. 2022;17(5):e0268272. doi: 10.1371/journal.pone.0268272 35560168 PMC9106162

